# Four eras of geriatric femoral neck fracture surgery: a bibliometric analysis of half-century research evolution and emerging frontiers

**DOI:** 10.3389/fsurg.2026.1894817

**Published:** 2026-08-28

**Authors:** Da-Wei Zhang, Hai-Ting Hong, Liang-He Lin, Zhen-Xin Fu

**Affiliations:** Department of Orthopedics, Shishi City Hospital, Quanzhou, Fujian, China

**Keywords:** arthroplasty, bibliometric analysis, CiteSpace, femoral neck fracture, geriatric, internal fixation, VOSviewer

## Abstract

**Background:**

The core clinical decision in geriatric femoral neck fracture—internal fixation vs. arthroplasty—has remained contested for half a century. Although landmark trials such as FAITH, HEALTH, and WHiTE 5 have profoundly reshaped clinical practice, no bibliometric study has systematically mapped the complete research architecture and knowledge evolution trajectory of this field from 1975 to the present. This study aimed to characterize the global research landscape and knowledge evolution of surgical treatment for geriatric femoral neck fracture using a tri-tool framework integrating CiteSpace, VOSviewer, and Bibliometrix with dual-database validation (WoSCC + Scopus).

**Methods:**

The WoSCC and Scopus databases were searched in parallel on May 24, 2026, with no date restriction. WoSCC yielded 2,147 documents (1,906 articles and 241 reviews); Scopus yielded 6,019 documents (5,689 articles and 330 reviews). After DOI-based deduplication, approximately 6,592 unique records remained. WoSCC served as the primary analytical dataset, with Scopus providing independent multi-database validation. Bibliometrix was used for descriptive statistics, VOSviewer for keyword co-occurrence and country collaboration networks, and CiteSpace for keyword/reference burst detection, co-citation clustering, and timeline analysis.

**Results:**

(1) Annual publications grew from 1 in 1975 to 203 in 2025. (2) The USA and China constitute the first-tier publishing nations (1,029 vs. 1,020 publications), yet China's average citations per article (11.5) lag substantially behind those of the United Kingdom and Sweden. (3) Bhandari M (43 publications) and Frihagen F (33 publications) were the most prolific authors; *Injury* was the top journal (144 publications). (4) Keyword burst analysis revealed a four-era knowledge evolution with distinct burst signatures: Foundational Era (1991–2001, *femur* 26.22), Fixation Debate Era (2002–2013, *internal-fixation* 15.28), Landmark Trial Era (2014–2018), and Perioperative Medicine Era (2019–present, *femoral neck system* 10.74). (5) The strongest citation bursts were the HEALTH trial (NEJM, strength 33.85) and Keating 2006 (JBJS Am, 29.19).

**Conclusions:**

This field has undergone four paradigm shifts from biomechanical description to perioperative comprehensive management. Although China ranks among the top-tier publishing nations alongside the USA, its research paradigm must transition from technique-oriented toward patient-outcome-oriented and multicenter evidence-based approaches to bridge the persistent citation impact gap.

## Introduction

Geriatric femoral neck fracture represents the most surgically complex type among osteoporotic fractures. Approximately 1.6 million new cases occur annually worldwide (1, 2). The core surgical dilemma lies in the vulnerable blood supply of the femoral head, which confers persistently high risks of avascular necrosis and nonunion following internal fixation, while arthroplasty, although eliminating these risks, introduces new complications such as dislocation and periprosthetic fracture (3).

The debate surrounding “internal fixation vs. arthroplasty” and “total hip vs. hemiarthroplasty” has generated a substantial body of clinical evidence over the past half century. Parker et al. (2002) provided the first Level I evidence from a single-center RCT demonstrating that hemiarthroplasty yielded significantly lower reoperation rates than internal fixation (4). Tidermark et al. (2003) further compared internal fixation with total hip arthroplasty (5). Between 2017 and 2020, three multicenter RCTs were published in rapid succession: FAITH (Fixation using Alternative Implants for the Treatment of Hip fractures, Lancet, *n* = 1,079) (6), HEALTH (Hip Fracture Evaluation with Alternatives of Total Hip Arthroplasty vs. Hemiarthroplasty, NEJM, *n* = 1,495) (7), and HIP ATTACK (Hip fracture Accelerated surgical TreaTment And Care tracK, Lancet, *n* = 2,970) (8). The WHiTE 5 (Warwick Hip Trauma Evaluation 5) trial established the superiority of cemented over uncemented hemiarthroplasty (9), and its causal forest analysis further propelled the field toward individualized medicine (10).

Prior bibliometric studies have either focused on single subtopics such as total hip arthroplasty and internal fixation (11, 12), covered hip fractures broadly without surgical specificity (13), or examined artificial intelligence applications in hip fractures (14). No bibliometric paper has yet characterized the geriatric femoral neck fracture surgery literature as a system anchored by the IF vs. HA vs. THA decision axis and covering the full evolutionary trajectory of the field. This study aimed to employ a CiteSpace + VOSviewer + Bibliometrix tri-tool framework, based on the full-time-span WoSCC dataset (1975–2026), to systematically map the research landscape and knowledge evolution of this field across half a century.

## Methods

### Study design and reporting

**Figure 1:**
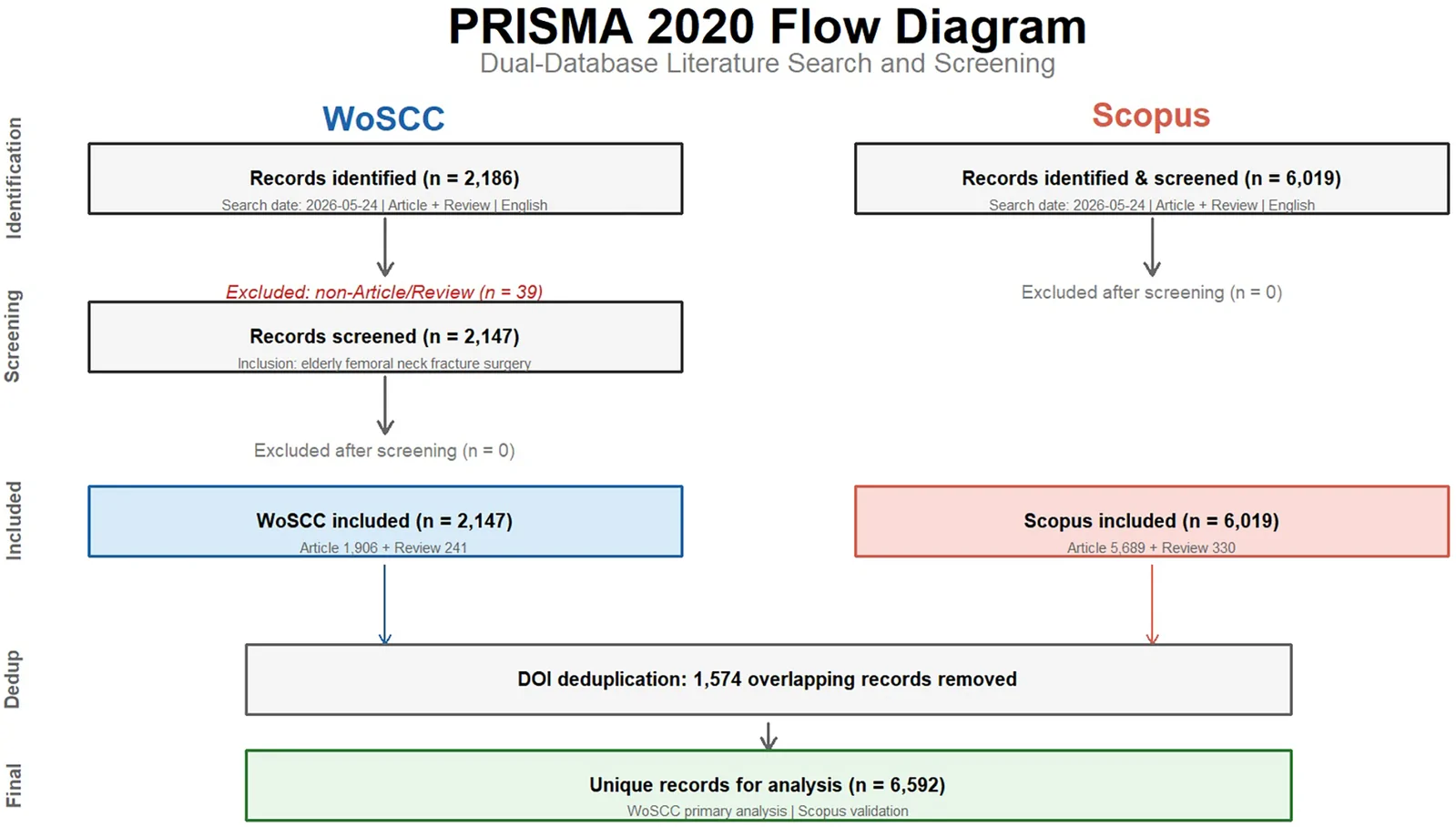
PRISMA flow diagram (dual-database). WoSCC: 2,186 records identified, 39 excluded, 2,147 included (1,906 articles, 241 reviews). Scopus: 6,019 records identified and screened, 0 excluded, 6,019 included (5,689 articles, 330 reviews). After DOI deduplication (1,574 overlapping records), 6,592 unique records were retained for analysis.

This study was designed as a systematic bibliometric review. The PICOS framework was defined as: Population — geriatric patients with femoral neck fracture; Intervention — any surgical treatment (internal fixation, hemiarthroplasty, total hip arthroplasty, or variants); Comparison — not applicable (descriptive bibliometric mapping); Outcome — publication and citation patterns, keyword trends, and thematic evolution; Study design — original articles and reviews. The completed PRISMA 2020 checklist and the PRISMA 2020 flow diagram (Figure 1) are provided.

### Search strategy

The Web of Science Core Collection (WoSCC) and Scopus were used as dual data sources. Both searches were conducted on May 24, 2026, with no time-span restriction.

**WoSCC search:** TS = (“femoral neck fracture*” OR “intracapsular hip fracture*” OR “intracapsular femoral fracture*” OR “subcapital fracture*” OR “transcervical fracture*”) AND TS* *=* *(elderly OR geriatric OR “older adult*” OR “older patient*” OR “aged” OR “aging”) AND TS* *=* *(surg* OR operat* OR arthroplast* OR hemiarthroplast* OR “total hip arthroplasty” OR “total hip replacement” OR “internal fixation” OR “cannulated screw*” OR “sliding hip screw” OR “dynamic hip screw” OR osteosynthesis).

**Scopus search:** TITLE-ABS-KEY of the same logical structure.

Both databases were limited to Article and Review document types, English language. Following the PRISMA 2020 flow diagram (Figure 1), WoSCC retrieved 2,186 records with 39 excluded, yielding 2,147 documents (1,906 articles and 241 reviews). Scopus retrieved 6,019 documents (5,689 articles and 330 reviews). After DOI-based deduplication, 1,574 records were found in both databases, yielding approximately 6,592 unique records. WoSCC served as the primary dataset for all main analyses and visualizations because CiteSpace, which performed the core analytical methods including keyword burst detection, co-citation clustering, and timeline visualization, natively supports only the WoS plain-text export format. The Scopus dataset was therefore used for independent multi-database validation rather than as the primary analytical source. This constraint is widely acknowledged in bibliometric research. To further assess whether the choice of primary database could influence the major conclusions, all core bibliometric indicators (annual publication trends, top countries, leading journals, and keyword frequencies) were independently extracted from Scopus and cross-compared with the WoSCC results (Section 3.11).

Regarding the age definition of the elderly population: since the databases do not support precise filtering by patient age, this study employed elderly-related keywords (elderly, geriatric, aged, etc.) in the search. The threshold of ≥60 years was adopted in accordance with the World Health Organization's definition of older persons in developing countries and consistent with the age cutoff commonly used in hip fracture literature (15). Two authors independently screened the titles and abstracts of all retrieved records. Disagreements were resolved through discussion with a third reviewer. Given that not all publications explicitly report patient age in the title or abstract, studies that met all other inclusion criteria but lacked explicit age information were retained, as the elderly-specific keyword strategy inherently enriched the search for the target population. This pragmatic approach, while maximizing sensitivity, represents a limitation acknowledged in Section 4.5.

### Analysis tools

- **CiteSpace 6.4.R2** (16): Keyword and reference burst detection, co-citation clustering, timeline visualization, and dual-map overlay. Parameters: time slicing 1975–2026 (2026 data up to the search date), 1 year per slice; node selection strategy: g-index with scale factor k = 25. The g-index was chosen over the top-N threshold because it favors highly cited items while still including lower-citation nodes that contribute structural connectivity, yielding a more representative network for a field with heterogeneous citation patterns such as geriatric hip fracture surgery. Pathfinder network pruning and merged network pruning were disabled to preserve the full linkage structure. The four-era evolutionary framework was delineated based on the temporal clustering of keyword burst onset and offset years identified by CiteSpace's burst detection algorithm, with era boundaries placed at natural inflection points where multiple burst terms simultaneously emerged or terminated.
- **VOSviewer 1.6.20** (17): Keyword co-occurrence networks, co-occurrence clustering, density visualization, country collaboration networks, and temporal overlay visualization.
- **Bibliometrix 4.3.0** (R 4.6.0) (18): Descriptive statistics and thematic evolution analysis.

### Data preprocessing and evaluation metrics

Prior to analysis, a thesaurus file was loaded in VOSviewer and an alias list (citespace.alias) was configured in CiteSpace to unify hyphenation variants, singular/plural forms, and spelling discrepancies. The h-index was used to measure academic impact, and citation burst strength was employed to identify keywords or references exhibiting sharp citation increases within specific time windows. CiteSpace cluster labeling used the log-likelihood ratio (LLR) algorithm. For country-level publication counts, because Bibliometrix automatically splits U.S. addresses into 261 records at the state/city level and U.K. addresses into constituent countries (England, Scotland, Wales, Northern Ireland) when parsing the all-author C1 field, the present study aggregated these records manually before ranking. The study was conducted in accordance with the PRISMA 2020 statement for systematic reviews (19).

## Results

### Annual publication output

**Figure 2:**
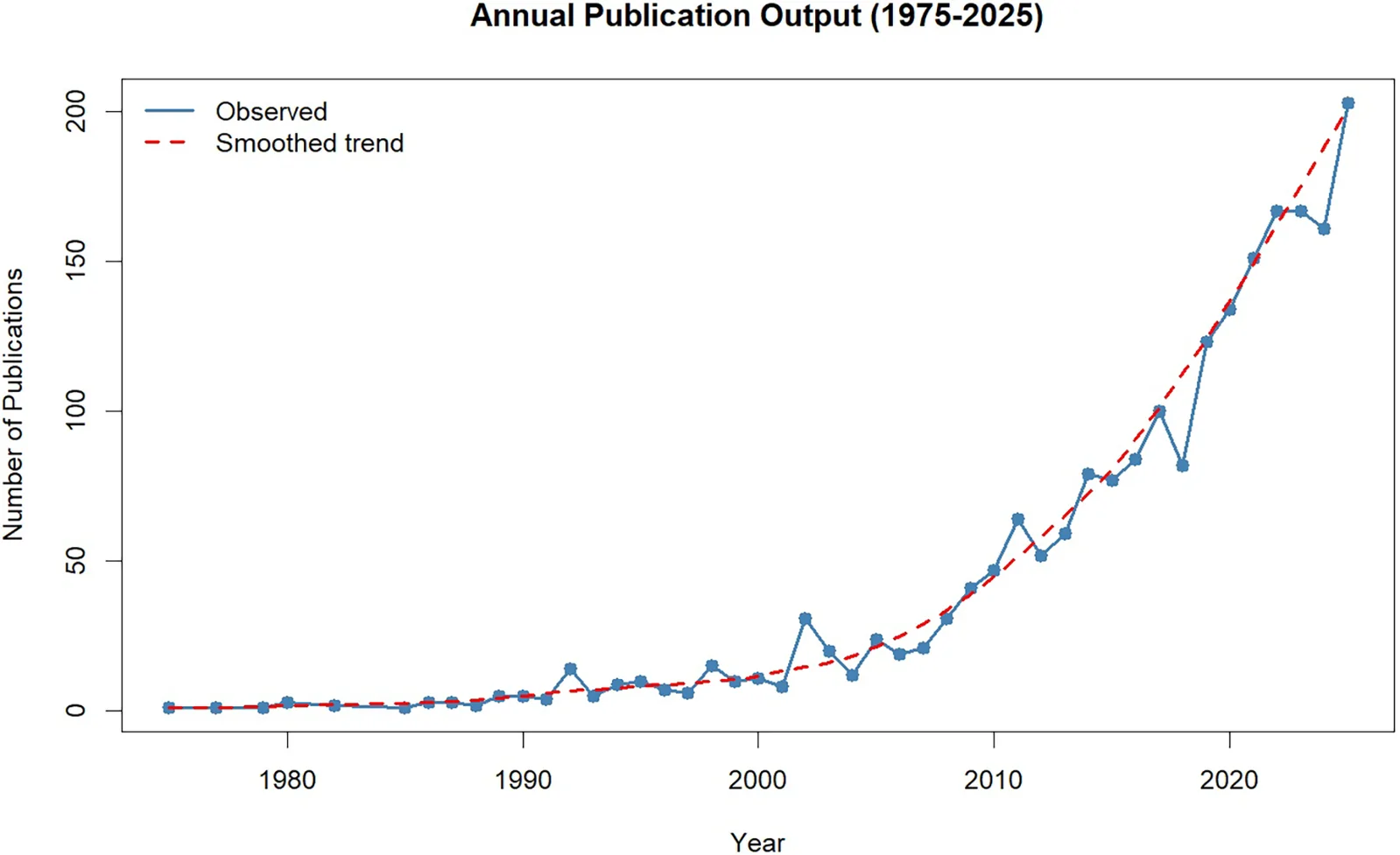
Annual publication output (1975–2025). Blue line: actual publication count; red line: LOWESS trend line. Data for 2026 are incomplete and not included.

The 2,147 documents spanned 1975 to 2026. Annual publications increased from 1 in 1975 to 203 in 2025 (Figure 2). The growth trajectory exhibited four phases: a sporadic output period (1975–1990, mean 1.7 publications/year), a slow growth period (1991–2005, mean 12.4/year), an accelerated growth period (2006–2015, mean 49.0/year), and a rapid expansion period (2016–2025, mean 137.2/year). A notable dip occurred in 2018 (82 publications, an 18% decline from 100 in 2017), followed by sustained growth from 2019 to 2025. The year 2026 was incomplete (72 publications as of the search date) and was excluded from trend analysis.

### Country and institutional distribution

**Figure 3:**
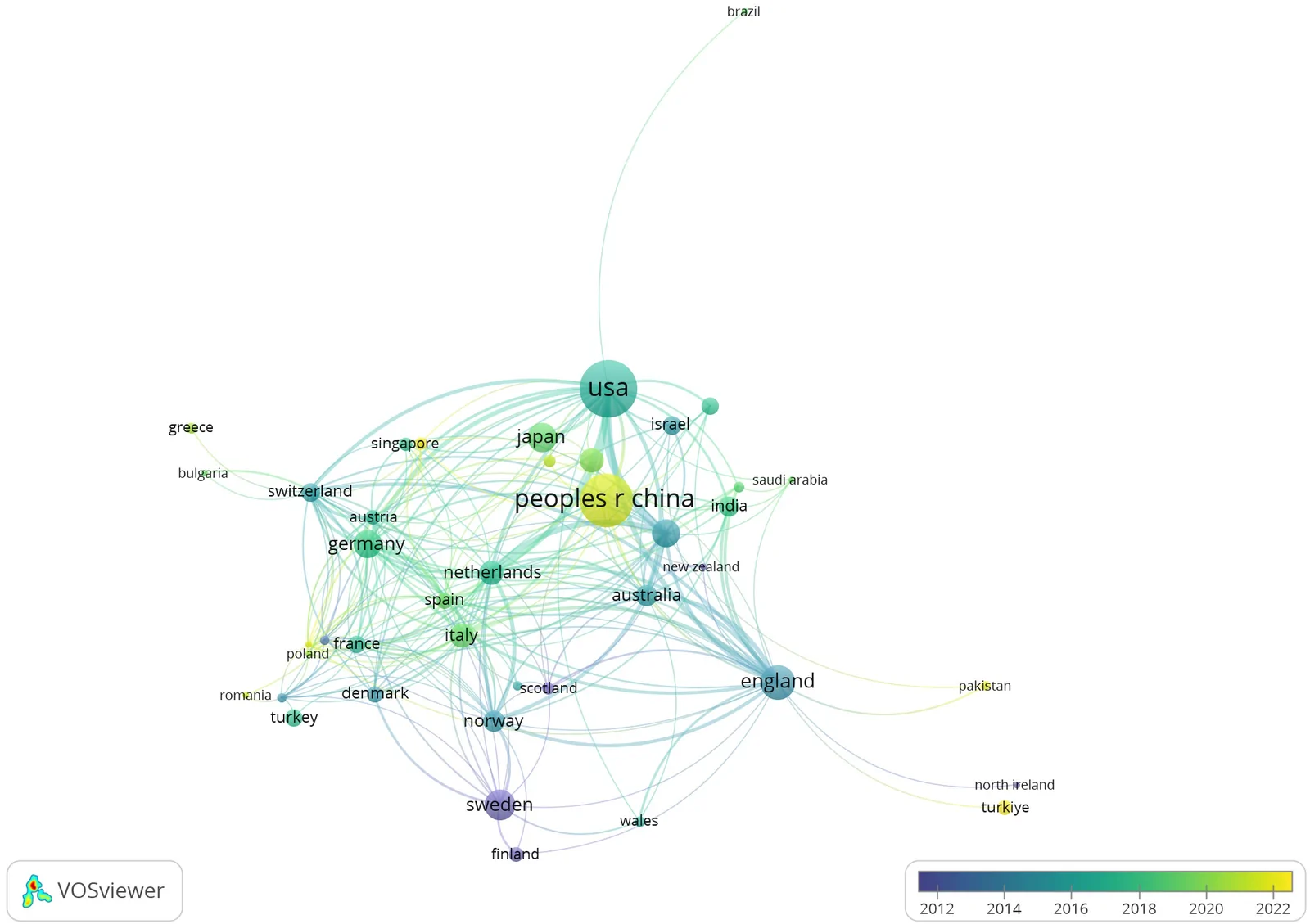
Temporal overlay of the country collaboration network. Node size represents publication volume; link thickness represents collaboration strength. Color indicates average year of research activity (blue = earlier, yellow = later).

The USA (1,029 publications) and China (1,020 publications) were closely matched, together contributing nearly half of the global output (Table 1), consistent with the macro-trend of Sino-American dominance in global publishing across medical fields (20). Citation impact showed an inverse gradient: China's average citations per article were only 11.5, substantially lower than those of the United Kingdom, Sweden, and other traditional research powerhouses. The temporal overlay of the country collaboration network (Figure 3) revealed a multipolar structure. Despite China's leading publication volume, its international collaboration link strength (15 links) was lower than that of the United Kingdom (32 links) and the USA (28 links). In addition, China's mean publication year (2021.2) was significantly later than those of the United Kingdom (2014.6) and the USA (2016.5), reflecting the concentration of Chinese research activity in recent years.

**Table 1:** Top 12 countries by publication volume.

Rank	Country	Publications
1	USA	1,029
2	China	1,020
3	United Kingdom	426
4	Japan	359
5	Sweden	324
6	Canada	284
7	Netherlands	251
8	South Korea	245
9	Germany	231
10	Australia	214
11	Italy	214
12	Norway	201

### Comparison of research focus: China vs. USA–UK

**Figure 4:**
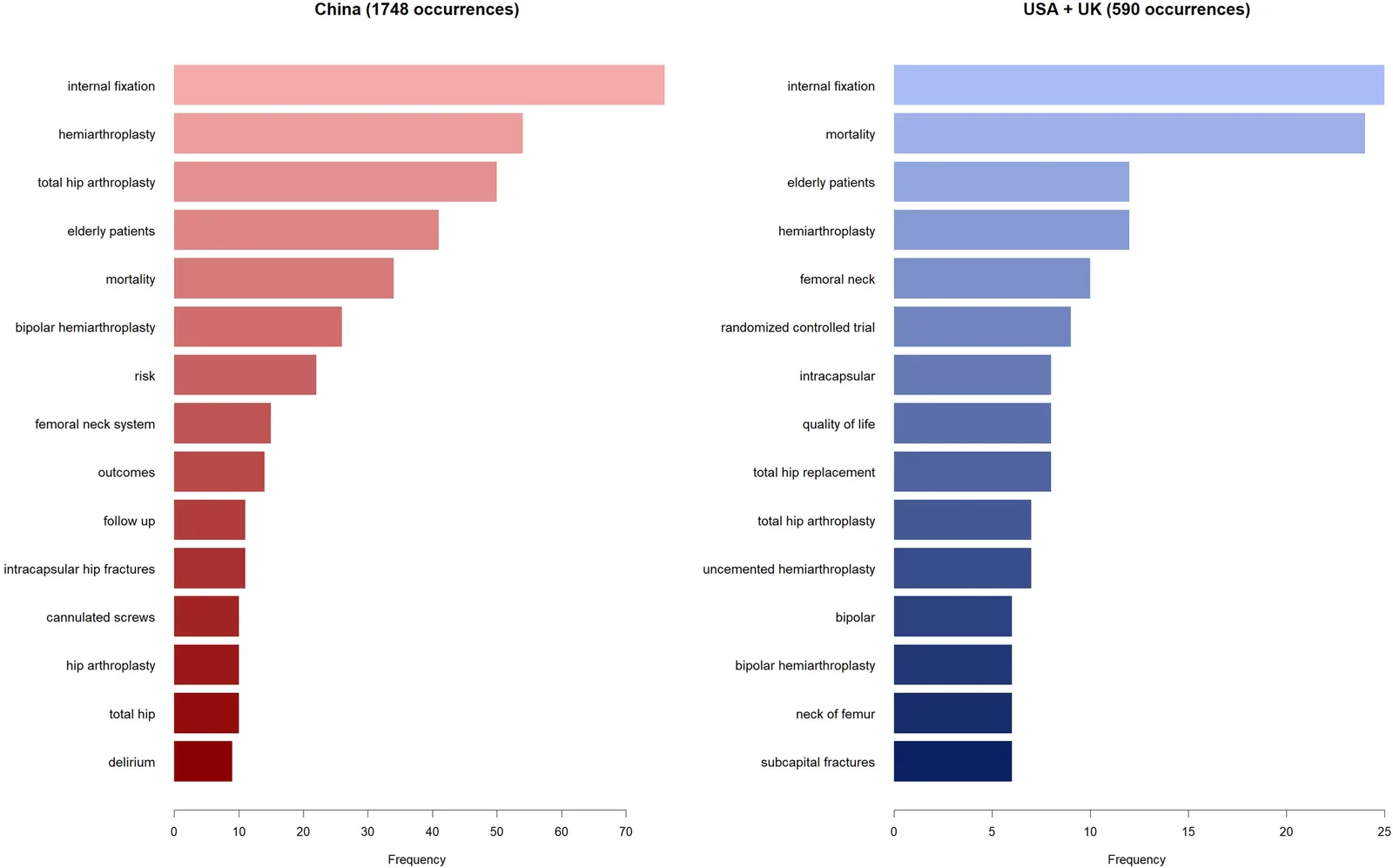
Comparison of high-frequency keywords: China vs. USA + UK. Left: China; Right: USA + UK. “Occurrences” refers to the total frequency of keywords extracted from the corresponding country's publications, not the number of publications.

Keywords from Chinese (1,020 publications) and USA–UK publications were independently extracted and compared (Figure 4). Both shared core terms such as *internal fixation*, *hemiarthroplasty*, and *mortality*. However, USA–UK keywords included patient-centered outcome terms such as *randomized controlled trial* and *quality of life*, whereas Chinese keywords were dominated by technique-oriented terms. This focus divergence provides a structural explanation for the citation gap.

### Prolific authors and core journals

The top five most prolific authors were Bhandari M (43 publications), Frihagen F (33), Sprague S (29), Rogmark C (25), and Schemitsch EH (23) (Table 2). Bhandari M also held the highest total citation count, to which his 2017 NEJM review (Table 4, #2), the FAITH trial published in Lancet the same year (6), and the 2019 HEALTH trial (7) collectively contributed. *Injury* was the most productive journal (144 publications) (Table 3).

**Table 2:** Top 15 most prolific authors.

Rank	Author	Publications	h-index
1	BHANDARI M	43	18
2	FRIHAGEN F	33	19
3	SPRAGUE S	29	12
4	ROGMARK C	25	14
5	SCHEMITSCH EH	23	12
6	TIDERMARK J	22	20
7	BZOVSKY S	21	9
8	PONZER S	20	18
9	MUKKA S	19	11
10	POOLMAN RW	18	9
11	GUSTAFSON Y	16	15
12	SLOBOGEAN GP	16	11
13	GJERTSEN JE	15	13
14	PARKER MJ	15	11
15	SKÖLDENBERG O	15	11

**Table 3:** Top 12 core journals.

Rank	Journal	Publications	h-index
1	Injury	144	33
2	BMC Musculoskeletal Disorders	79	21
3	Journal of Arthroplasty	77	22
4	Archives of Orthopaedic and Trauma Surgery	68	25
5	Journal of Orthopaedic Trauma	68	24
6	Geriatric Orthopaedic Surgery & Rehabilitation	63	16
7	Journal of Orthopaedic Surgery and Research	51	20
8	Clinical Orthopaedics and Related Research	47	33
9	Acta Orthopaedica	43	26
10	International Orthopaedics	42	21
11	Journal of Bone and Joint Surgery-American Volume	37	23
12	Journal of Clinical Medicine	33	7

**Table 4:** Top 10 highly cited publications.

Rank	First Author	Title	Journal	Year	Citations
1	Abrahamsen B	Excess mortality following hip fracture	Osteoporos Int	2009	836
2	Bhandari M	Management of acute hip fracture (Review)	NEJM	2017	504
3	Bhandari M	Internal fixation vs. arthroplasty	JBJS Am	2003	436
4	Bhandari M	Operative management	JBJS Am	2005	366
5	Smith T	Pre-operative indicators for mortality	Age Ageing	2014	318
6	Dyer CB	Postoperative delirium	Arch Intern Med	1995	317
7	Keating JF	Randomized comparison of reduction	JBJS Am	2006	312
8	Volkert D	ESPEN guideline	Clin Nutr	2022	309
9	Kammerlander C	Ortho-geriatric service	Osteoporos Int	2010	278
10	Peeters CMM	Quality of life after hip fracture	Injury	2016	276

### Keyword co-occurrence network and thematic clusters

**Figure 5:**
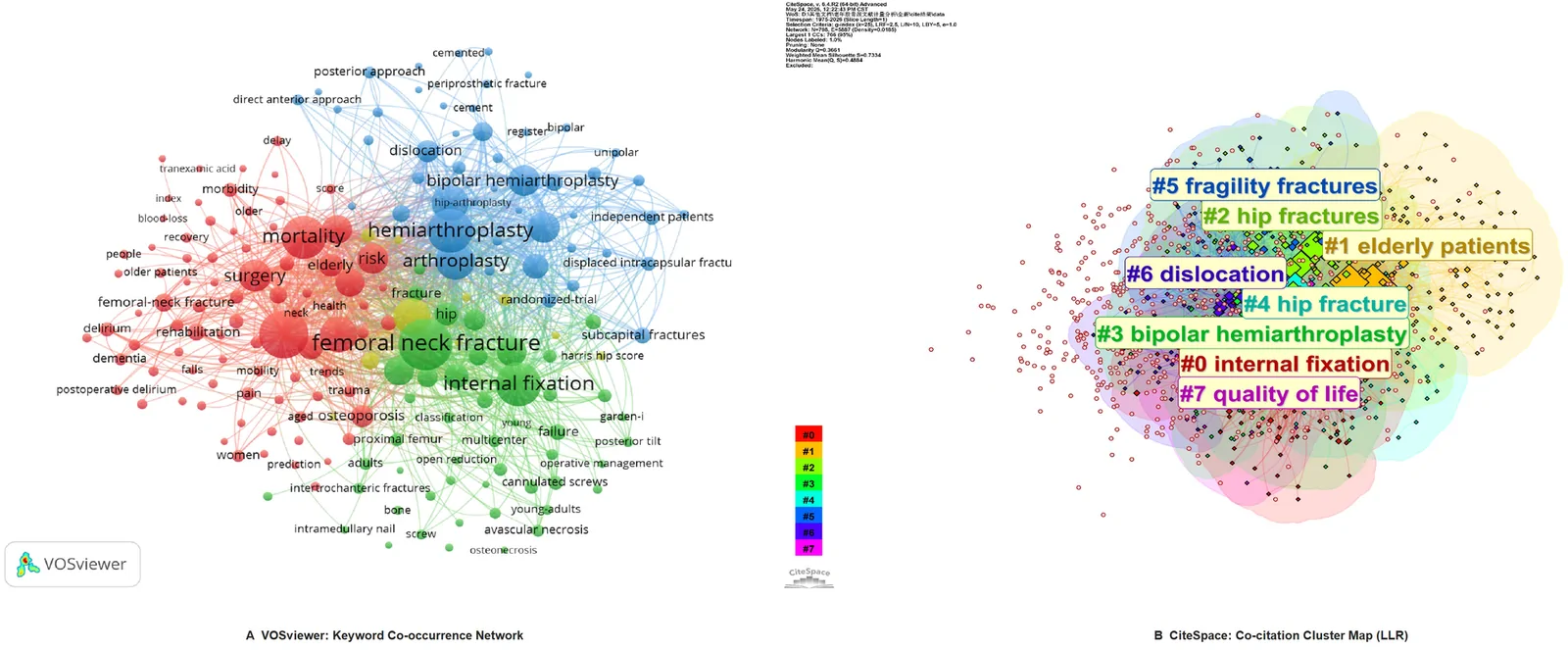
Keyword co-occurrence network and co-citation clusters. **(A)** VOSviewer keyword co-occurrence network: node size represents keyword frequency; colors distinguish clusters (red = perioperative complications, gree*n* = internal fixation techniques, blue = arthroplasty comparison, yellow = long-term outcomes); link thickness represents co-occurrence strength. **(B)** CiteSpace co-citation cluster map: cluster labels are automatically extracted by the LLR algorithm; smaller cluster numbers (#0, #1, …) indicate larger cluster sizes.

VOSviewer keyword co-occurrence clustering identified four major thematic clusters (Figure 5A): Cluster 1 (red, 84 nodes) — perioperative complications and mortality, with core keywords *mortality*, *risk factors*, *anemia*, and *postoperative delirium*; Cluster 2 (green, 48 nodes) — internal fixation techniques and implant selection, with core keywords *internal fixation*, *cannulated screws*, and *sliding hip screw*; Cluster 3 (blue, 48 nodes) — arthroplasty modality comparison, with core keywords *hemiarthroplasty*, *total hip arthroplasty*, *cemented*, and *uncemented*; Cluster 4 (yellow, 10 nodes) — specific fracture types and long-term outcomes. CiteSpace co-citation clusters (Figure 5B) showed strong concordance with VOSviewer clusters on major themes, providing cross-validation between independent tools.

### Keyword burst detection and four-era evolution

**Figure 6:**
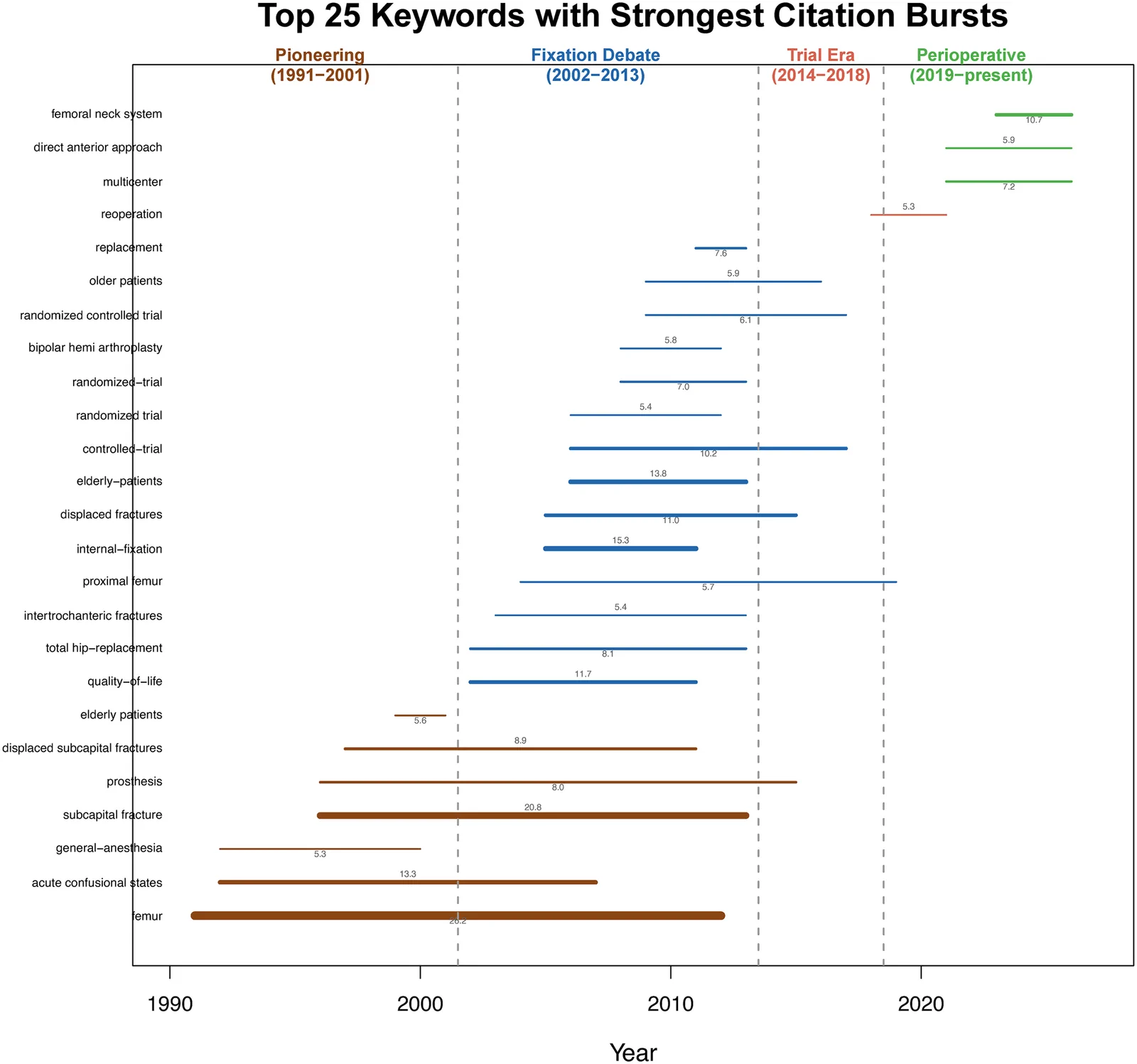
Keyword burst analysis (Top 25). Red bar segments represent burst duration; blue segments represent non-burst periods. The horizontal axis represents time; keywords are ordered by burst start year in descending order.

CiteSpace identified 25 keywords with significant burst signals (Figure 6). Their temporal distribution exhibited a clear four-phase pattern:

**1991–2001 (Foundational Era):** *femur* (strength 26.22, the strongest burst keyword in the entire dataset), *subcapital fracture* (20.80), *acute confusional states* (13.26), and *prosthesis* (8.02) — predominantly anatomical and prosthetic material terms — underwent concentrated bursts, reflecting the field's origin in descriptive surgical technique. Among these, *subcapital fracture* (1996–2013) spanned both the Foundational and Fixation Debate eras, representing the longest-lasting burst keyword in the dataset.
**2002–2013 (Fixation Debate Era):** *internal-fixation* (15.28), *elderly-patients* (13.79), *quality-of-life* (11.66), *total hip-replacement* (8.12), and *controlled-trial* (10.21) underwent concentrated bursts, marking the transition from technical description to comparative effectiveness evaluation.
**2014–2018 (Landmark Trial Era):** FAITH (2017) and HEALTH (2019) catalyzed sustained bursts of evidence-based methodological terms, as research shifted from single-center experience to multicenter RCT evidence synthesis.
**2019–present (Perioperative Medicine Era):** *femoral neck system* (10.74), *multicenter* (7.23), and *direct anterior approach* (5.84) underwent concentrated bursts, signaling a shift toward individualized perioperative management and novel implant evaluation. Among these, *femoral neck system* (2023–2026) was the strongest technology-specific burst of this era, reflecting the emergence of the Femoral Neck System (FNS) as a novel internal fixation device attracting current research attention.

### Thematic evolution

**Figure 7:**
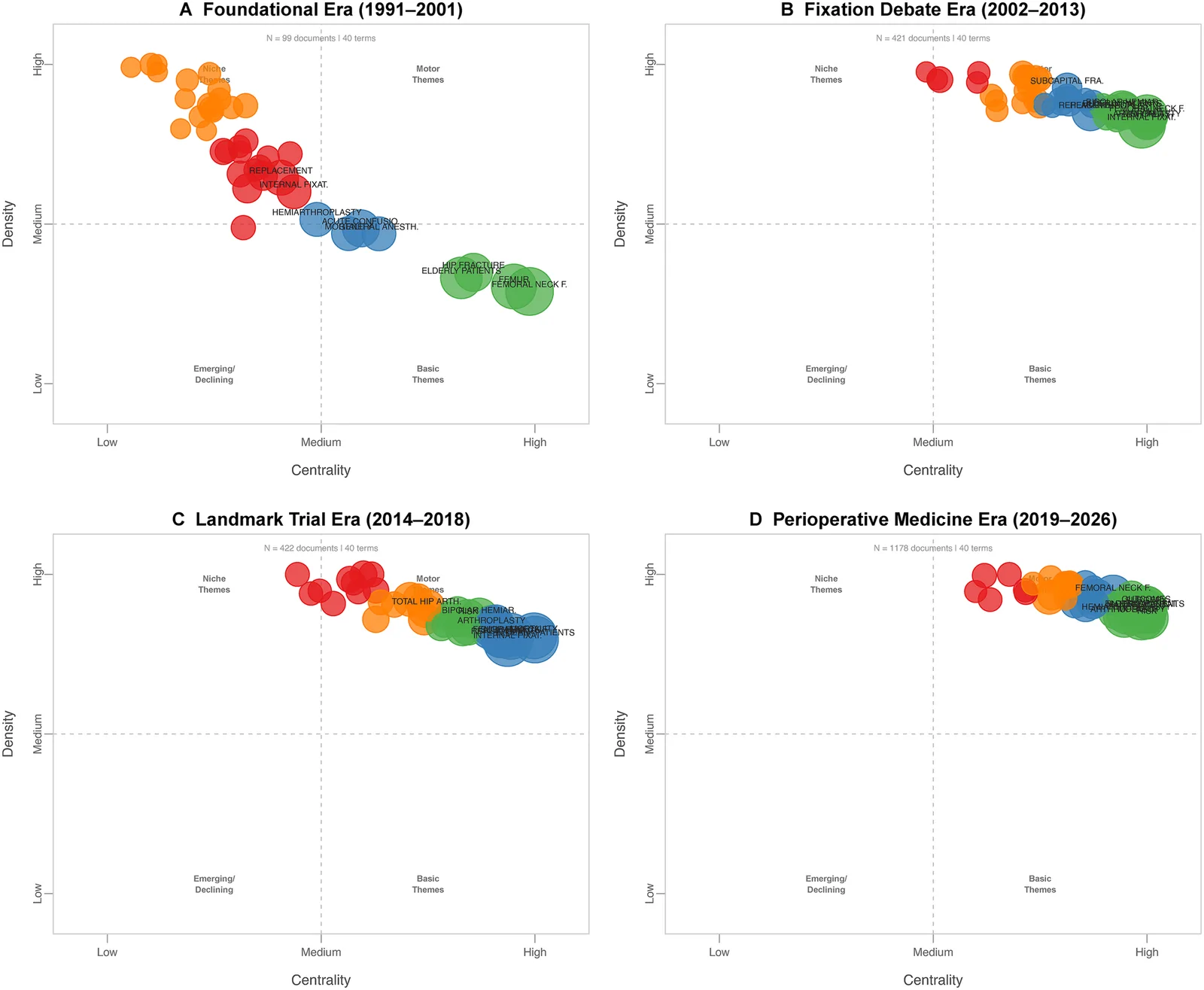
Four-era thematic strategic diagrams. The *x*-axis represents Callon's Centrality; the *y*-axis represents Callon's Density. Each point represents a theme; point size reflects the number of keywords within that theme. Upper-right: Motor Themes; lower-right: Basic Themes; upper-left: Niche Themes; lower-left: Emerging or Declining Themes. **(A)** Foundational Era (1991–2001). **(B)** Fixation Debate Era (2002–2013). **(C)** Landmark Trial Era (2014–2018). **(D)** Perioperative Medicine Era (2019–2026).

Thematic strategic diagrams (Figure 7) plotted each era's keywords by Callon's centrality (*x*-axis) and density (*y*-axis), partitioning them into four quadrants: upper-right — Motor Themes (high centrality, high density); lower-right — Basic Themes (high centrality, low density); upper-left — Niche Themes (low centrality, high density); lower-left — Emerging or Declining Themes (low centrality, low density). Cross-era comparison revealed the following: the Foundational Era (1991–2001, Figure 7A) featured themes concentrated on basic descriptions of osteoporosis and fracture healing, with a limited number of motor themes; the Fixation Debate Era (2002–2013, Figure 7B) exhibited rapid thematic diversification, with internal fixation, arthroplasty, and mortality entering the motor quadrant; the Landmark Trial Era (2014–2018, Figure 7C) saw randomized controlled trials and meta-analysis emerge as independent themes; the Perioperative Medicine Era (2019–2026, Figure 7D) displayed the densest thematic network, with FNS, direct anterior approach, and postoperative delirium emerging as new themes, marking the paradigm shift from single-modality comparison to comprehensive perioperative management.

### Citation burst and co-citation timeline

Citation burst analysis revealed a multi-phase knowledge evolution trajectory (Tables 4, 5). In the Foundational Era, Keating 2006 (JBJS Am, strength 29.19) (7) was the earliest strong citation burst. In the Fixation Debate Era, Bhandari 2003 (JBJS Am) led with 436 citations, while Burgers 2012 (Int Orthop, 16.65) and others advanced systematic comparisons of internal fixation and arthroplasty. In the Landmark Trial Era, HEALTH (Bhandari 2019, NEJM, 33.85) was the strongest citation burst in the entire dataset. In the Perioperative Medicine Era, WHiTE 5 (Fernandez 2022, NEJM, 16.87) achieved near-zero-delay citation explosion.

**Table 5:** Top 15 citation bursts.

Rank	Burst Reference	Strength	Period
1	Bhandari 2019, NEJM — HEALTH	33.85	2020–2024
2	Keating 2006, JBJS Am	29.19	2006–2011
3	Blomfeldt 2007, JBJS Br	26.05	2008–2012
4	Blomfeldt 2005, JBJS Am	19.45	2006–2010
5	Dolatowski 2019, JBJS Am	18.62	2020–2024
6	Rogmark 2002, JBJS Br	17.83	2002–2007
7	Lewis 2019, J Arthroplasty	17.50	2020–2024
8	Baker 2006, JBJS Am	17.16	2008–2011
9	Parker 2010, Cochrane	16.99	2011–2015
10	Fernandez 2022, NEJM — WHiTE 5	16.87	2022–2026
11	Veronese 2018, Injury	16.74	2021–2023
12	Taylor 2012, JBJS Am	16.71	2013–2017
13	Burgers 2012, Int Orthop	16.65	2015–2017
14	Bhandari 2017, JBJS Am	16.50	2019–2022
15	Florschutz 2015, J Orthop Trauma	16.33	2018–2022

Of the top 10 highly cited publications, Abrahamsen 2009 (rank 1, *n* = 836 citations) and Bhandari 2017 (rank 2, *n* = 504, published as a Review article) are not original surgical studies. Their high citation counts reflect their status as foundational reference works that have shaped the prognostic framework within which surgical decisions are made. The remaining eight publications are original studies or systematic reviews directly addressing surgical management.

### Co-citation timeline

**Figure 8:**
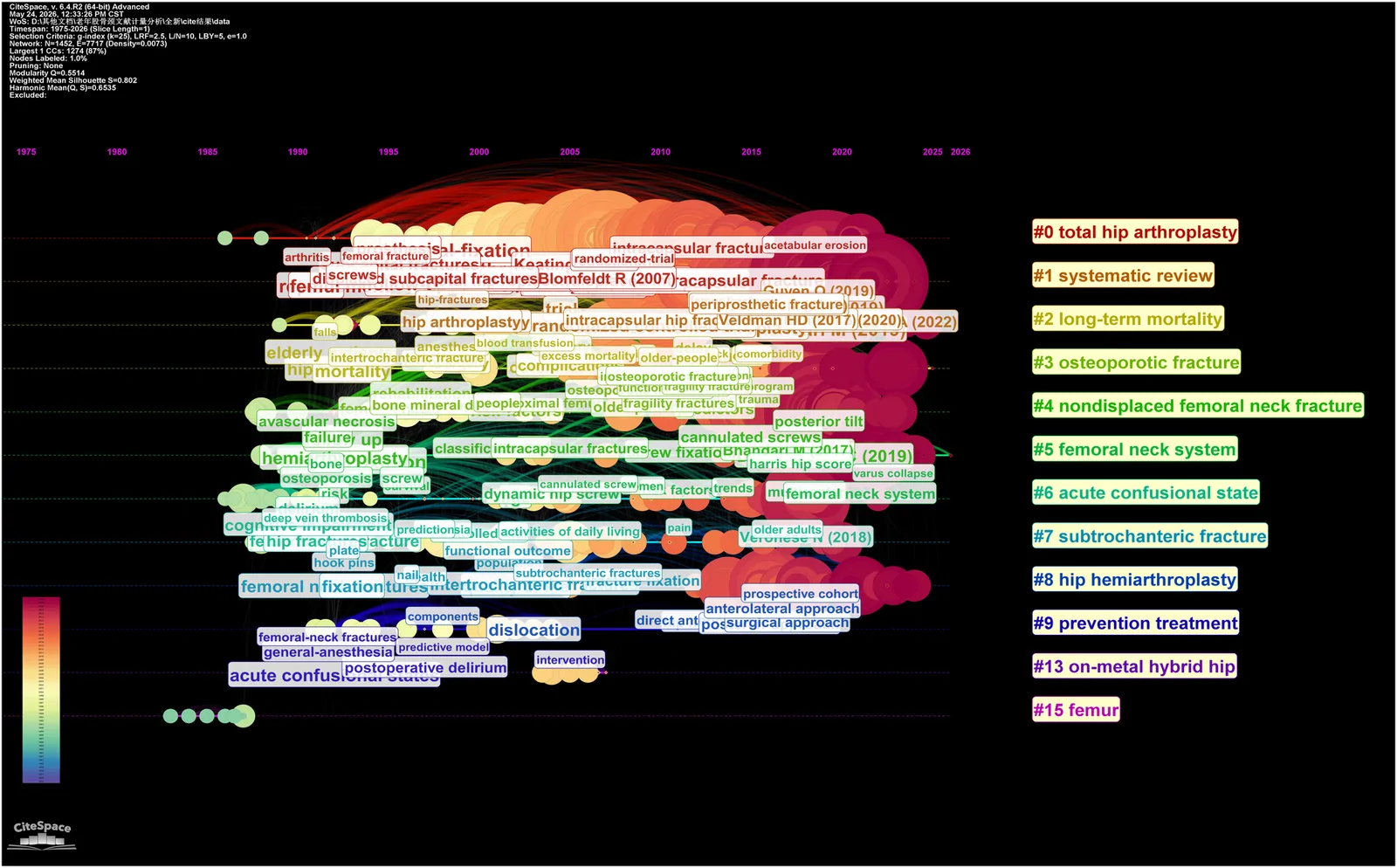
Co-citation timeline. The horizontal axis represents time; each horizontal line represents a co-citation cluster.

The co-citation timeline (Figure 8) visually demonstrated the diachronic migration of the knowledge base from fracture fixation techniques to perioperative management, with early clusters focused on anatomical reduction and internal fixation materials and recent clusters centered on postoperative delirium, multidisciplinary co-management, and prognostic prediction.

### Dual-map overlay

**Figure 9:**
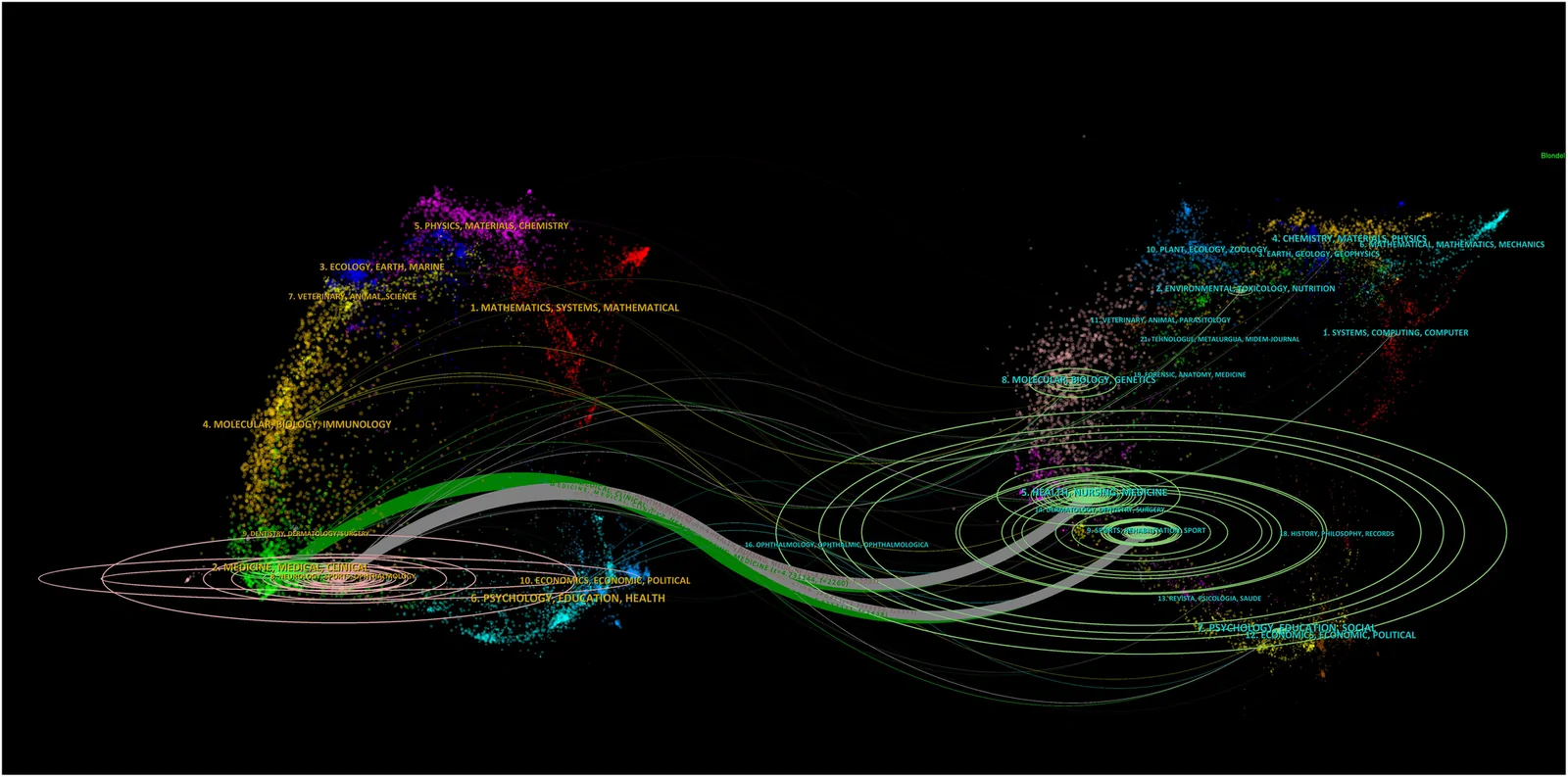
Dual-map overlay. Left: citing journals; Right: cited journals. Colored arcs represent citation trajectories.

The dual-map overlay (Figure 9) delineated two major citation pathways: (1) orthopedic/surgery journals → clinical medicine and geriatric medicine journals; (2) internal medicine/geriatric medicine journals → epidemiology and public health journals. The bridging between the two pathways strengthened markedly after 2020, reflecting the disciplinary convergence of orthopedics and geriatric medicine.

### Multi-database validation

To evaluate the robustness of single-database bibliometric conclusions, an independent search using an identical strategy was performed in Scopus (6,019 documents; 1975–2026). Core metrics were extracted and cross-compared with the WoSCC results (Table 6). The annual publication trends showed near-perfect correlation between the two databases (r = 0.99, *p* < 0.001; Supplementary Figure S1), both exhibiting the same four-phase growth pattern. The top five countries were identical in ranking (USA, China, United Kingdom, Japan, Sweden), with Scopus showing systematically higher absolute counts due to its broader journal coverage. Journal rankings were similarly robust: *Injury* ranked first in both databases (WoSCC: 144; Scopus: 468), while *Journal of Arthroplasty* and *BMC Musculoskeletal Disorders* appeared in the top 10 of both. Core keywords—*femoral neck fracture*, *hemiarthroplasty*, *total hip arthroplasty*, and *mortality*—occupied concordant high-frequency positions across databases, confirming the stability of the thematic cluster structure. These findings confirm that the principal conclusions derived from the WoSCC primary analysis—including the four-era evolutionary framework, the country-level citation gradient, and the core thematic architecture—remain robust and consistent under cross-database validation, effectively ruling out single-database selection bias as a confound for the observed patterns.

**Table 6:** Multi-database validation.

Metric	WoSCC	Scopus
Documents included	2,147	6,019
Time span	1975–2026	1975–2026
Top 1 country (pubs)	USA (1,029)	USA (2,553)
Top 2 country	China (1,020)	China (2,281)
Top 1 journal	Injury (144)	Injury (468)
Top 1 keyword	femoral neck fracture	femoral neck fracture

## Discussion

The core clinical question in geriatric femoral neck fracture surgery—internal fixation or arthroplasty—has remained unchanged for half a century. Yet the evidence ecosystem surrounding this question has evolved from fragmented single-center experience into a multi-level, multicenter evidence-based network. Based on a bibliometric analysis of 2,147 WoSCC publications with independent Scopus validation (6,019 records), this study provides the first data-driven, complete characterization of this evolutionary trajectory. The parallel Scopus analysis confirmed near-perfect inter-database consistency in annual publication trends (r = 0.99; Supplementary Figure S1), country rankings, and core journal distributions, demonstrating that the four-era evolutionary framework and the observed citation patterns are robust to single-database selection bias.

### From “Fracture Healing” to “Patient Benefit”—clinical transformation across four eras

In the research landscape of the Foundational Era (1991–2001), the two anatomical terms *femur* (26.22, the strongest burst keyword in the dataset) and *subcapital fracture* (20.80) occupied the absolute center. This was no coincidence. The dominant belief in 1990s orthopedic practice was that fracture reduction quality determined prognosis. Research focused almost exclusively on biomechanical aspects—screw placement technique, the mechanical advantages of sliding hip screws, and protection of femoral head blood supply. The literature of this period was dominated by cadaveric studies and finite element analyses; clinical studies were predominantly single-center retrospective case series.

The keyword landscape of the Fixation Debate Era (2002–2013) revealed a sustained academic contest spanning over a decade. *internal-fixation* (15.28) and *elderly-patients* (13.79) burst in parallel, while *quality-of-life* (11.66) entered the high-frequency burst list for the first time—researchers began to address a more fundamental question: fracture healing does not equal patient benefit. Parker (2002) published a 455-patient single-center RCT in JBJS Br, providing the first Level I evidence in response to this proposition: the reoperation rate of hemiarthroplasty was significantly lower than that of internal fixation. Tidermark (2003) further compared internal fixation with total hip arthroplasty. Contemporaneously, pivotal RCTs such as Keating 2006 (JBJS Am, burst strength 29.19) and Blomfeldt 2007 (JBJS Br, 26.05) formed the core evidence pillars of this era (Table 5).

The Landmark Trial Era (2014–2018) was defined by a structural transformation. Although HEALTH (2019) and HIP ATTACK (2020) were published after the nominal end of this era, their design, patient enrollment, and methodological philosophy were products of the multicenter RCT paradigm shift that characterized this period; they are therefore discussed here as the culmination of this era rather than as belonging exclusively to the subsequent perioperative phase. Prior to this period, orthopedic clinical research was dominated by single-center RCTs and systematic reviews—evidence was abundant but fragmented. FAITH (2017, *n* = 1,079) and HEALTH (2019, *n* = 1,495) redefined the evidence hierarchy: multicenter, multinational, large-sample RCTs replaced single-center experience as the new evidence standard. HIP ATTACK (2020, *n* = 2,970) (8) further introduced the concept of accelerated surgery into geriatric hip fracture perioperative management, a strategy further supported by recent evidence (21). HEALTH, with a burst strength of 33.85, ranked first among all citation bursts in this study (Table 5), reflecting the entire field's urgent demand for high-quality evidence.

The Perioperative Medicine Era (2019–present) is characterized not by the rise of a particular surgical modality but by an overall shift in research focus. The bursts of *multicenter* (7.23) and *direct anterior approach* (5.84) indicate that the research question has expanded from “which procedure is superior” to “for which patient, at which time point, with which perioperative management strategy.” The proliferation of keywords such as postoperative delirium, multidisciplinary co-management, nutritional support, and machine learning prognostic models marks the paradigm transition from “orthopedic surgery” to “orthogeriatric medicine.” Yet, even in the Perioperative Medicine Era, the core debate between internal fixation and arthroplasty remains unresolved—Metcalfe et al. (2025) recently noted in their review that the optimal treatment for displaced femoral neck fractures remains an open clinical question (22).

### Delayed burst—citation dynamics of clinical trials reveal a field's cognitive maturation

The citation burst data in Table 5 reveal a phenomenon previously unreported in bibliometric studies: landmark clinical trials do not trigger citation explosions immediately upon publication but exhibit measurable delay periods. HEALTH (2019) did not show a burst signal until 2020 (1-year delay); WHiTE 5 (2022) achieved near-zero-delay explosion.

From a clinical perspective, this delay reflects the collective learning curve of the global orthopedic community. When HEALTH was published in 2019, multicenter hip fracture RCTs remained an unfamiliar form of evidence for most orthopedic surgeons—for the preceding two decades, evidence production had been dominated by single-center studies and Cochrane systematic reviews. The 1-year delay in HEALTH's citation burst represents the time window required for the field to absorb and trust a new evidence paradigm.

By the time WHiTE 5 was published in 2022, the academic ecosystem had fundamentally changed. HEALTH had established cognitive groundwork and trust in multicenter hip fracture RCTs—demonstrating that such trials could generate conclusions directly altering clinical practice. The question WHiTE 5 answered—whether to use cemented or uncemented fixation in hemiarthroplasty—is among the core daily decisions in orthopedic trauma. Its conclusion (cemented fixation superior to uncemented) was directly incorporated into NICE guidelines, influencing the most common procedural choice worldwide. From evidence paradigm establishment to clinical behavior change, HEALTH required 1 year and WHiTE 5 approached zero—the compression of this time differential itself quantifies the growth in a field's evidence-based maturity.

### FNS: when technology outruns evidence

*femoral neck system* (FNS, strength 10.74, 2023–2026) was the third-strongest keyword burst signal of the Perioperative Medicine Era. Yet, no FNS-related RCT appeared among the Top 10 highly cited publications (Table 4) or Top 15 citation bursts (Table 5). This contradictory pattern of “strong keyword burst + zero citation burst” points to a clinically recognizable phenomenon—technology adoption preceding high-quality evidence.

FNS is currently undergoing rapid global uptake, but its evidence base derives primarily from biomechanical studies and single-center case series. This trajectory closely parallels the expansion pattern of locking plates in the 2000s: widespread clinical adoption based on mechanical reasoning and early clinical impressions in the absence of RCT evidence. Bibliometric analysis here demonstrates a unique early-warning capability—it can objectively identify technical domains where the speed of clinical adoption exceeds the speed of evidence accumulation. For clinical departments evaluating the introduction of FNS, this signal carries reference value.

### China's “High Volume, Low Impact” orthopedic research: not a language issue, but a research-question issue

China, with 1,020 publications, is virtually tied with the USA at 1,029; together the two nations contribute nearly half of global output. Yet China's average of only 11.5 citations per article falls significantly behind traditional research powerhouses such as the United Kingdom and Sweden. What underlies the shortfall in the citation impact of Chinese research?

The country-level keyword comparison (Figure 4) provides a data-driven explanation. China's Top 15 keywords are dominated by technique-oriented terms (*internal fixation*, *femoral neck system*, *cannulated screws*), whereas USA–UK keywords are led by patient-outcome and evidence-based methodological terms such as *randomized controlled trial*, *quality of life*, and *survival*. This is not a matter of language proficiency or database coverage—it is a systematic divergence at the level of research paradigm.

A further structural factor is temporal lag. China's mean publication year of 2021.2 is approximately five years later than those of the United Kingdom (2014.6) and the USA (2016.5) (Figure 3 temporal overlay coloring). This means that Chinese research output is heavily concentrated in the academic maturity phase of the field—a large volume of papers published at a stage when international collaboration networks and citation hierarchies have already solidified, imposing a longer time window for late entrants to embed themselves into existing high-citation chains. However, with China's sustained high output after 2020, this temporal gap is narrowing.

The consequences of this divergence are clear. The technical details provided by Chinese literature—screw placement strategies, internal fixation mechanical analyses, finite element simulations—have engineering value, but they answer “how to do it,” not “which strategy is better.” What the international orthopedic community most urgently needs is precisely the latter—evidence that can directly guide clinical decision-making. The three trials FAITH, HEALTH, and HIP ATTACK together enrolled over 5,500 patients, none of whom contributed randomized data from mainland China—a fact more telling than citation metrics alone.

The path to bridging this gap is not simply to increase the volume of English-language publication or to standardize citation practices. High-volume Chinese orthopedic centers need to substantively embed themselves in multicenter RCT collaborative networks—to include their own patients within randomization frameworks and to extend their research agenda from “technique refinement” to “clinical outcomes.” This matters not only for citation metrics but, more importantly, for generating locally relevant evidence to support clinical decision-making for China's elderly hip fracture patients.

### Limitations and future directions

This study used WoSCC as the primary data source. Although an independent Scopus search (6,019 records) was conducted for multi-database cross-validation (Section 3.11), confirming the robustness of the core conclusions, databases such as PubMed and Dimensions, as well as non-English literature, were not included, which may underestimate the academic output of certain regions. The year 2026 is incomplete and was excluded from trend analysis. Although the keyword-based search strategy maximizes enrichment for elderly populations, its ability to discriminate age thresholds is inherently limited, and the title/abstract screening step may have excluded relevant studies that did not explicitly report patient age in their abstracts. In addition, bibliometrics reflects publication patterns rather than clinical practice patterns; conclusions such as “FNS technology adoption preceding evidence” warrant cautious interpretation—citation patterns in the published literature are a mirror of clinical reality, not clinical reality itself.

Future research can advance along two directions. First, bibliometric signals for novel internal fixation systems such as FNS should be cross-referenced against real-world clinical registry data to verify whether “technology adoption preceding evidence” is a genuine clinical phenomenon. Second, advancing the paradigm migration of Chinese orthopedic research from “technique-oriented” to “patient-outcome-oriented” requires substantive changes initiated at the study design stage: multicenter, prospective, with patient-reported outcomes as endpoints.

## Conclusions

(1) **Four-Era Evolutionary Framework:** Foundational Era (1991–2001) → Fixation Debate Era (2002–2013) → Landmark Trial Era (2014–2018) → Perioperative Medicine Era (2019–present), an objective periodization grounded in dual-source CiteSpace burst data.
(2) **Delayed Burst Pattern of Landmark Trials:** Landmark trials exhibit a 0–1 year citation burst delay; the zero-delay burst of WHiTE 5 (9) is attributable to a mature evidence-based ecosystem.
(3) **FNS Technology Adoption–Evidence Gap:** FNS shows a keyword burst of 10.74 but lacks corresponding RCT citation support, identifying a domain where clinical technology adoption outpaces high-quality evidence accumulation.
(4) **East–West Citation Gap:** China focuses on surgical technique; the USA and UK focus on clinical outcomes—bridging the gap requires substantive Chinese participation in international multicenter platform trials.

## Data Availability

Publicly available datasets were analyzed in this study. This data can be found here: "The data are available from the Web of Science Core Collection (https://www.webofscience.com) and Scopus (https://www.scopus.com). These are subscription-based databases; no accession numbers apply. All data can be retrieved by reproducing the search strategies detailed in Section 2.1 of the Methods."
