# Supplementary material for: Four eras of geriatric femoral neck fracture surgery: a bibliometric analysis of half-century research evolution and emerging frontiers
Source: Front Surg. 2026 Aug 28;13:1894817. doi: 10.3389/fsurg.2026.1894817 (PMC13564913; doi:10.3389/fsurg.2026.1894817)
Supplement: Supplementary file 1 [file Table1.docx]

# PRISMA 2020 Checklist

| Section | Item | Reported on |
| --- | --- | --- |
| **Title** | 1. Identify as systematic review | Page 1, Title |
| **Abstract** | 2. Structured summary | Page 1, Abstract |
| **Rationale** | 3. Rationale for review | Introduction |
| **Objectives** | 4. Explicit objectives | Introduction, last paragraph |
| **Eligibility criteria** | 5. Inclusion/exclusion criteria | Methods 2.1 |
| **Information sources** | 6. Databases searched (WoSCC, Scopus) | Methods 2.1 |
| **Search strategy** | 7. Full search strategy for both databases | Methods 2.1 |
| **Selection process** | 8. Screening process | Methods 2.1; Fig. 1 |
| **Data collection** | 9. Data extraction methods | Methods 2.2 |
| **Data items** | 10. Outcomes (publications, citations, bursts) | Methods 2.3 |
| **Study risk of bias** | 11. Not applicable (bibliometric mapping) | Limitations 4.5 |
| **Effect measures** | 12. Bibliometric indices defined | Methods 2.3 |
| **Synthesis methods** | 13. CiteSpace/VOSviewer/Bibliometrix | Methods 2.2 |
| **Certainty assessment** | 14. Cross-database validation (r=0.99) | Results 3.11 |
| **Study selection** | 15. PRISMA flow diagram | Fig. 1 |
| **Study characteristics** | 16. Bibliometric data cited | Results |
| **Risk of bias** | 17. Not applicable | Limitations 4.5 |
| **Results synthesis** | 18. For each outcome | Results 3.1-3.11 |
| **Reporting biases** | 19. Cross-database validation | Results 3.11 |
| **Certainty of evidence** | 20. Limitations discussed | 4.5 |
| **Discussion** | 21-22. Summary + limitations | 4.1-4.5 |
| **Registration** | 23. Not registered (bibliometric review) | N/A |
| **Protocol** | 24. Protocol not prepared (descriptive mapping) | N/A |
| **Support** | 25. Funding statement | Declarations |
| **Competing interests** | 26. Declared | Declarations |
| **Data availability** | 27. WoSCC repository | Declarations |
